# Analysis of 22,655 presentations with back pain to Perth emergency departments over five years

**DOI:** 10.1186/1865-1380-4-59

**Published:** 2011-09-17

**Authors:** Michael T Lovegrove, George A Jelinek, Nicholas P Gibson, Ian G Jacobs

**Affiliations:** 1 Department of Emergency Medicine, Joondalup Health Campus, Shenton Road, Joondalup, 6027, WA, Australia; 2 Discipline of Emergency Medicine, University of Western Australia, Nedlands, WA, Australia; 3 Department of Emergency Medicine, Sir Charles Gairdner Hospital, Nedlands, WA, Australia

## Abstract

**Background:**

Back pain is a significant cause of disability in the community, but the impact on Emergency Departments (EDs) has not been formally studied. Patients with back pain often require significant time and resources in the ED.

**Aims:**

To examine the characteristics of patients presenting with back pain to the ED, including final diagnosis, demographics of those attending and temporal distribution of presentations.

**Methods:**

Emergency presentations in the metropolitan area of Perth, Western Australia, for 2000-2004 were searched using a linked database covering all the major hospitals (Emergency Care Hospitalisation and Outcome Study database). All presentations with the triage code for back pain were extracted and analysed.

**Results:**

A total of 22,655 presentations with back pain were identified, representing 1.9% of total presentations. Simple muscular or non-specific back pain accounted for only 43.8% of presentations, with other causes such as renal colic and pyelonephritis accounting for the majority. The young (<15 years old) and elderly (>75 years old) were more likely to have non-muscular causes for their back pain. Muscular back pain presentations occurred mostly between 0800 and 1600, with high proportions presenting on the weekends. Patients with simple muscular back pain spent a mean of 4.4 h in the ED, representing a significant outlay of resources.

**Conclusion:**

Back pain has a significant impact on EDs, and staff should be alert for another pathology presenting as back pain. There is a need for multidisciplinary back pain teams to be available 7 days a week, but only during the day.

## Introduction

Back pain is a common problem and has a large effect on work productivity, mental health and physical activities. Approximately 80% of Australians suffer back pain at some time, and two thirds have some form of back pain in a 12-month period [1]. These findings are similar in other regions [2-5]. Back pain places strain on the health system with as many as 50% of sufferers still having problems at 5 years [6]. If initial treatment is effective, especially by a multidisciplinary team, then the recurrence rate and long-term disability can be reduced [2,7-9]. Determining when these teams, which usually consist of paramedical staff such as physiotherapists and occupational therapists, can be of the most use would assist in resource allocation.

Only a minority (45%) of those with back pain seek help [10]. About 10% have significant disability [1], and a proportion of those present to the emergency department (ED) for care. As with most emergency presentations, the patient arrives with an undefined complaint that requires diagnosis. Back pain can be relatively benign, but can also be a symptom of more serious illness.

No large study looking at the pattern of ED back pain presentations by adults or the range of diagnoses presenting as back pain to the ED has been published. One study reported children presenting with back pain over a 1-year period and identified 225 presentations, most due to acute trauma, although infections such as urinary tract infections and viral illnesses were also found [11].

## Objective

The objective of this study was to identify patients presenting to EDs in Perth, Western Australia, with back pain and to characterise the pattern of their presentations. Hospital discharge and subsequent mortality were also examined.

## Methods

### Data sources

The Emergency Care Hospitalisation and Outcome Study (ECHO) database [12], which is a database that follows patients through admission and subsequent presentations, was used.

The primary data were collected using EDIS (Emergency Department Information Systems, version 10.0, Health Administration Solutions, Sydney), which contains information on emergency presentations including patient demographics, arrival and discharge dates and times, presenting complaints, mode of arrival and triage score, and disposition and discharge diagnosis from all of the metropolitan EDs in Perth. Hospital admission data were obtained from the Hospital Morbidity Dataset. Death records were obtained from the Western Australian Mortality Database.

### Data analysis

EDIS records, hospital admission records and the Mortality Database were linked by the Western Australian Data Linkage Unit using probabilistic matching. Data analysis was performed with the Statistical Package for the Social Sciences (SPSS, version 12.0, Chicago, IL) and Microsoft Excel for characteristics of patients, clinical problems, mortality rates and departmental factors. To calculate mortality rates, the last ED presentation of each patient in the dataset elicited cumulative mortality figures at 2 and 30 days.

### Back pain study

The ECHO database was searched for the period 1 July 2000 to 31 December 2004.

Presentations where the triage nurse selected the triage code for "back pain" were extracted and analysed. This cohort represents patients who, at the time of their first assessment in the ED, indicated that back pain was the most important reason for their presentation.

The patients' age, sex, time of presentation (by day of week and time of day), diagnosis (at discharge from the ED and from hospital if admitted) and outcome of their time in the hospital system, including admission, re-presentations and mortality, were extracted. Data regarding their time in the ED and treatment time (time from being seen by a doctor to time of discharge) were also extracted.

This study group was then divided into two subgroups based on the diagnosis made by the ED doctor. The first group had a diagnosis consistent with non-specific pain or muscular injury, with conditions likely to be benign. The second group of patients consisted of those with conditions likely to be "non-muscular" in nature such as renal colic, pyelonephritis and pancreatitis. A decision was made to include radicular pain and vertebral fractures in this second group as the management of these conditions is often different from simple soft tissue back pain. The two groups were analysed and compared, looking for significant differences in presentation or patient characteristics.

### Ethics approval

Ethical and record linkage approvals were granted by the Human Research Ethics Committee at the University of Western Australia and the Confidentiality of Health Information Committee of Western Australia.

## Results

A total of 1,171,713 presentations to Perth EDs were recorded between the 1 July 2000 and 31 December 2004. Of these, 22,655 (1.9%) were coded with "back pain" as the primary complaint. Table 1 summarises the results for all back pain presentations, the "muscular group" and the "non-muscular" group.

**Table 1:** 

	All back pain	Muscular	Non-muscular
Total	22655	9,926 (43.8%)	12,729 (56.2%)
Female	51.4%	50%	53%*
Most common ages	35-44 years	35-44 and 25-34 years	35-44 and 25-34 years
Average age	46.3	46.6	46.2
Most common days presenting	Sunday and Monday	Sunday and Monday	Sunday and Monday
% presenting on weekends	30.2%	30.3%	30.2%
Admitted	25.4%	17.1%	31.8%*
Admission LOS	6.0 days	6.4 days*	5.8 days
ED time	4.8 h	4.4 h	5.1 h*
Treatment time	3.6 h	3.1 h	4.1 h*
Death in ED	10	1	9*
Death within 48 h	30	4	26*
Death within 30 days	263	75	188*

*Significant.

**Figure 1:**
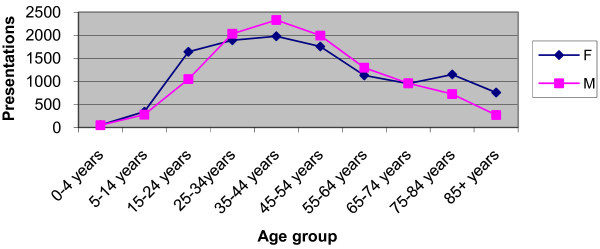
**Presentations by Gender and Age Group**.

The most common age group was between 35 and 44 (Figure 1). Females presented in significantly larger numbers in the older age groups (75 years and over) and also in adolescence (15-24) (p < 0.005) (Figure 1).

Patients presented most often on Sundays with a gradual decrease in presentations until Friday before increasing on Saturday. A slightly disproportionately higher number of presentations occurred on weekends, with 30.2% of back pain presentations occurring on Saturday and Sunday.

**Figure 2:**
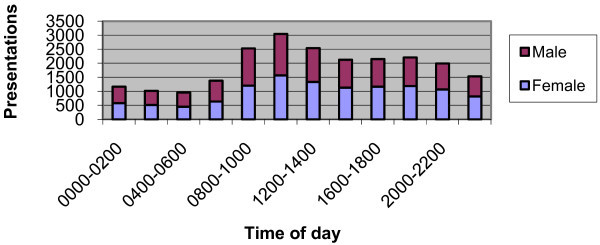
**Presentations by Gender and Time of Day**.

Presentations were most common between 0800 and 1400, accounting for 35.8% of total presentations, but large numbers of presentations continued until midnight. Relatively few patients (13.8%) presented between midnight and 0600 (Figure 2).

Overall, a quarter of patients (5,747, 25.4%) were admitted, with females admitted more often than males (27.2% vs. 23.4%, p < 0.005).

The majority of patients (88.9%) presented only once. However 2,125 patients had multiple presentations, with one individual presenting 104 times with back pain during the data collection period. The mean number of presentations was 1.2 attendances, with 97% of patients presenting only once or twice.

When presentations were analysed by cause, 43.8% (9,926) were diagnosed as a muscular problem or non-specific back pain ("muscular" group). The remaining 56.2% (12,729) were diagnosed as due to other medical and surgical causes ("non-muscular" group). Of this group, 1,066 (8.4%) did not wait for treatment. "Did not wait" or similar applied to 610 of these at time of discharge with the remaining 456 having a diagnosis made, but not waiting for subsequent treatment. Males were significantly more likely to not wait for treatment (p < 0.005).

### "Muscular" group

Muscular causes of pain were relatively rare in children under the age of 15 (2.0%).

Presentations for muscular pain occurred predominantly during the hours 0800-1400 (39.4% of presentations), with nearly 50% occurring in the 8 h between 0800 and 1600.

Only 17.1% of people in the "muscular" group required admission, but those admitted remained in hospital an average of 6.4 days (SD 10.7 days). One person was hospitalised for 163 days.

### "Non-muscular" group results

**Figure 3:**
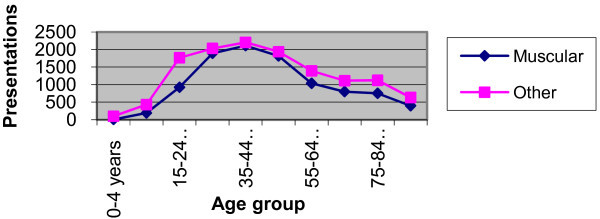
**Presentations by Category type and Age**.

In the "other" group, females represented 53% of the 12,729 presentations, significantly more than males (p < 0.005). The most common age groups remained the 25-34 and 35-44 groups, but the distribution of ages of those presenting was more spread, with much higher numbers in the 0-14 year age groups and 75+ age groups. Of 107 children below the age of 5 years who presented with back pain, 99 (92.5%) had a diagnosis other than muscular, and of those between the ages of 5 and 15, 69.1% (431 of 623 patients) were also in this group. Similarly, of those patients over 75 years of age presenting with back pain, 60.4% (1,756 of 2,904) had causes that were found to be non-muscular (Figure 3).

The admission rate for "non-muscular" category was 31.8%, significantly more than that for muscular causes (p < 0.005). However, those who were admitted had a mean length of stay of 5.8 days (SD 8.8 days), significantly less than those admitted for muscular causes (p < 0.005). The mean length of time spent in the ED was 5.1 h, 4.1 of these after being seen by the doctor. These times were significantly longer than for patients in the muscular group (p < 0.005).

There was significantly higher mortality at each time point (in ED, 2 days and 30 days after presentation) than for muscular back pain (p < 0.005).

The most common diagnoses made in the ED for those people in the "non-muscular" group were:

Renal colic (9.7%)

Sciatica/radiculopathy (8.0%)

Urinary tract infection or pyelonephritis (6.4%)

No disease found (2.6%)

### Hospital admissions

The most common hospital discharge diagnoses for all patients presenting with back pain who required admission were:

Back pain (18.8%)

Renal colic (13.7%)

Lumbar sprain (11.1%)

Pyelonephritis or urinary tract infection (6.6%)

Vertebral crush fracture (3.6%)

Abdominal pain (2.3%)

Intervertebral disc prolapse (2.3%)

Pneumonia (1.5%)

Back pain was the presenting complaint in 47 cases of angina and 24 cases of myocardial infarction. A total of 53 patients with pulmonary emboli presented with back pain, and 13 episodes of pancreatitis presented with back pain as the primary complaint.

Seventeen cases of dissecting aortic aneurysms were diagnosed in the ED in patients presenting with back pain. Eighteen ruptured abdominal aortic aneurysms (AAA) were diagnosed in the ED. There were five deaths within 48 h of presentation from these 18 patients, with 3 in the ED.

## Discussion

### Size of problem

In our study, back pain in the ED was a large problem, accounting for nearly 2% of attendances to the departments, each presentation requiring, on average, between 4.4 and 5.1 h of ED time. Average treatment times after seeing a doctor were between 3 and 4 h before discharge occurred. This is despite nearly 83% of people with simple muscular or non-specific back pain being discharged home. Given the relatively low triage score assigned to the patients presenting with muscular back pain, a treatment time of less than an hour might be anticipated; however the treatment time observed was more than three times longer. Given the high rate of discharge, this treatment time is likely to represent difficulty in achieving adequate analgesia and mobility. The duration of time spent in EDs for those admitted is rising with the increasing problem of access block.

The high rates of low back pain described, for all ages in community surveys [11,13], but not seen in ED presentations, supports the finding that the majority of patients do not seek medical care for their pain [10].

### Gender and age variation

Consistent with previous studies more females, and especially adolescent girls, show a greater incident of back pain [3-5,10,11,13,14]. More elderly females presented with back pain than men (1.9 times more, age >75, 2.8 times more >85). The increasing number of females living to older age may account for this. Sixty percent of diagnoses in this elderly group were of the "non-muscular" category.

Those at the extremes of age are also less likely to have a simple muscular or non-specific cause for their pain. This was most marked in the youngest age group, with more than 90% of those children aged 0-4 years of age diagnosed with other causes for their back pain, but the effect was clearly seen up to the age of 14, and also in patients over the age of 75.

A significant number of children presenting with pain in the back were found to have infective causes (viral illnesses, urinary tract infections, meningitis, osteomyelitis), although there was also a large number with torticollis or wry neck that had been coded as neck/back pain by the triage nurse. Even taking these into account, only 33 of 107 children aged 0-4 years of age had a muscular cause for their pain, meaning that nearly 70% of young children had non-muscular causes.

### Time and day of week of presentations

**Figure 4:**
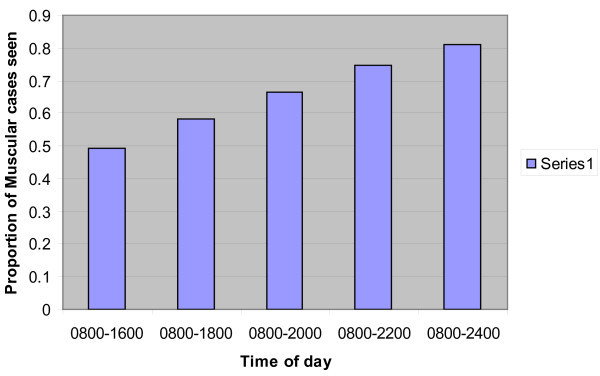
**Proportion of Muscular Back Pain seen by MDBP teams by rostered hours over 7 days per week**.

A significant number of patients present with back pain, particularly of a muscular or non-specific nature, on the weekends. This has relevance to acute back pain services where multidisciplinary back pain (MDBP) teams have been set up to assess and assist those with back pain. There is good evidence that these services will generate a quicker recovery of function [2,7-9], and our data suggest it would be beneficial to run these 7 days a week. By providing cover between 0800 and 1600, 7 days a week, 49.2% of cases could be seen. If this was extended to 1800 at night, 58.2% would be seen, and if extended through to midnight, 81.1% of cases could be seen (Figure 4). If the service is provided only between 0800 and 1600 on weekdays, then only 34.0% could be seen. By extending this to 1800 on weekdays, 40.0% could be seen, but even extending the service to midnight on weekdays, only 56.1% of cases could be seen.

Patients presenting with back pain after hours are more likely to be diagnosed with a non-muscular cause for the pain. Common alternate diagnoses were related to the renal tract, particularly renal colic. This would suggest that urinalysis, as a simple screening tool for urinary pathology, should be considered when there is doubt about the cause of back pain.

There was a surprisingly high mortality rate (1.2% 30 day mortality) for people presenting with back pain overall, and whilst most of this resulted from non-muscular causes, there was a 30-day mortality of 0.8% even in those with muscular or non-specific back pain. This may reflect that the event of pain is a marker of overall deteriorating function in some individuals, although this study is not capable of determining these underlying causes.

Ruptured abdominal aortic aneurysms and aortic dissections were rare (0.08% and 0.09% of presentations, respectively) when the initial complaint was one primarily of back pain, but given their gravity, should be considered in all cases of back pain, particularly in the elderly.

### Limitations

As with any retrospective analysis, this study is limited by the data entered by the treating doctors and nurses, although some of the variables used are not subject to data entry problems (including the patient demographics, presentation events and times). This study is likely to underestimate the frequency of presentation to ED with back pain as only patients with a triage code of "Back Pain" were selected. It also did not include those for whom back pain was present, but not considered the most important of their complaints by the triage nurse.

The division of the overall cohort (into "muscular" and "non-muscular" subgroups) was a relatively arbitrary decision, but enabled us to highlight more serious causes. A significantly larger number of people in the "non-muscular" category did not wait for treatment or left against advice, and this is likely to have prevented full assessment and, thus, an accurate diagnosis.

## Conclusion

This study has demonstrated that back pain as a primary complaint is a common problem in EDs, requiring a significant amount of resources. The majority of patients presenting with back pain had a cause other than a simple muscular pain or non-specific pain, and these presentations had a significant mortality. Patients who are elderly (>75 years of age) or young (<14 years of age) are more likely to have non-muscular causes for their pain and should receive more careful evaluation to look for serious pathology presenting as back pain.

This study also demonstrates a need for multidisciplinary teams managing muscular and non-specific back pain to be available 7 days a week up until the early evening (e.g. 1800 hours) to provide the most efficient coverage of people presenting with back pain of this type.

Education of staff or provision of analgesic and therapy guidelines or flowcharts may be of use in speeding up the management of muscular back pain in the ED.

Further studies looking at the efficacy of multidisciplinary back pain teams in reducing subsequent presentations or long-term morbidity associated with muscular back pain would be useful. A study looking at the causes of mortality in those presenting with back pain would also be of use in determining and treating preventable causes.

## Competing interests

The authors declare they have no competing interests

## Authors' contributions

ML was involved in study design, data anaysis, statistical analysis and manuscript preparation. GJ was involved in study design, data analysis and manuscript preparation. IJ was involved in study design, data analysis and manuscript preparation. NG was involved in study design, data extraction, data analysis and statistical analysis. All authors read and approved the final manuscript.

## Author information

Michael Lovegrove is an Emergency Specialist working in adult and paediatric Emergency Departments in Perth, Western Australia.
